# Epidemic Surveillance Using an Electronic Medical Record: An Empiric Approach to Performance Improvement

**DOI:** 10.1371/journal.pone.0100845

**Published:** 2014-07-09

**Authors:** Hongzhang Zheng, Holly Gaff, Gary Smith, Sylvain DeLisle

**Affiliations:** 1 Veterans Affairs Maryland Health Care System, Baltimore, Maryland, United States of America; 2 School of Medicine, University of Maryland, Baltimore, Maryland, United States of America; 3 Department of Biological Sciences, Old Dominion University, Norfolk, Virginia, United States of America; 4 School of Veterinary Medicine, University of Pennsylvania, Kennett Square, Pennsylvania, United States of America; Arizona State University, United States of America

## Abstract

**Backgrounds:**

Electronic medical records (EMR) form a rich repository of information that could benefit public health. We asked how structured and free-text narrative EMR data should be combined to improve epidemic surveillance for acute respiratory infections (ARI).

**Methods:**

Eight previously characterized ARI case detection algorithms (CDA) were applied to historical EMR entries to create authentic time series of daily ARI case counts (background). An epidemic model simulated influenza cases (injection). From the time of the injection, cluster-detection statistics were applied daily on paired background+injection (combined) and background-only time series. This cycle was then repeated with the injection shifted to each week of the evaluation year. We computed: a) the time from injection to the first statistical alarm uniquely found in the combined dataset (Detection Delay); b) how often alarms originated in the background-only dataset (false-alarm rate, or FAR); and c) the number of cases found within these false alarms (Caseload). For each CDA, we plotted the Detection Delay as a function of FAR or Caseload, over a broad range of alarm thresholds.

**Results:**

CDAs that combined text analyses seeking ARI symptoms in clinical notes with provider-assigned diagnostic codes in order to maximize the precision rather than the sensitivity of case-detection lowered Detection Delay at any given FAR or Caseload.

**Conclusion:**

An empiric approach can guide the integration of EMR data into case-detection methods that improve both the timeliness and efficiency of epidemic detection.

## Introduction

Epidemics of acute respiratory infections (ARI), whether due to influenza [1], [2], coronaviruses [3], [4], or other pathogens [5], [6], could overwhelm even the most developed health care systems. It is imperative to recognize these epidemics as early as possible, as the passage of time quickly degrades the effectiveness of mitigating measures [7].

Electronic data offer the opportunity for more timely and complete gathering of health information compared to what has historically been achieved through manual, paper-based reporting [8]. The increasingly rapid deployment of electronic medical records (EMR) [9] broadens the array of data that could be recruited for surveillance purposes [10], [11]. EMR-based surveillance could improve our response to a serious outbreak of ARI not only by allowing earlier recognition, but also by offering an efficient conduit for the information necessary to manage actual patients and to keep abreast of the evolving epidemic [12]–[14]. At present, however, the tantalizing potential of EMR-based surveillance remains in the making [15]–[18].

To gain insight on the conduct of surveillance in an EMR environment, we previously evaluated how EMR entries should be assembled to discover individuals with ARI [19]. We found that computerized free-text analyses aimed at uncovering ARI symptoms documented in outpatient clinical notes could complement diagnostic codes and other structured data to improve case detection. In this report, we asked if those EMR-enabled gains in case-detection could accelerate the discovery of ARI outbreaks. Using software to reconstitute a surveillance system operating prospectively on historical data sets, we compared alternative case-detection approaches for their ability to reduce the delay in detecting a modeled community outbreak of influenza. Our approach and results begin to chart how EMR-based information could be systematically organized to better serve public health surveillance.

## Methods

### Ethics Statement

The Institutional Review Boards of the Veterans Administration (VA) Maryland Health Care System and the University of Maryland approved this study. The study was granted a waiver of consent as risks were limited to information confidentiality and the work would not have otherwise been feasible, given the large number of EMR records screened for possible ARI. All EMR information was anonymized and de-identified prior to simulations and analyses, which used only daily case counts.

### Description of Procedures

#### EMR Data extractions and transformation

Historical EMR data were extracted from the Veterans Integrated Service Technology Architecture (VistA) repository using the MDE software (Strategic Reporting Systems Inc., Peabody, MA) and transferred to a Structured Query Language (SQL) relational database (SQL Server 2008, Microsoft Corp., Richmond, WA).

#### Background: authentic counts of patients with possible ARI

Outpatients with possible ARI were identified by applying previously developed ARI case-detection algorithms (CDAs) [19] to institutional databases derived from real historical EMR entries. ARI was defined as: positive respiratory virus culture/antigen OR any two of the following symptom, of no more than 7 days duration: a) cough; b) fever or chills or night sweats; c) pleuritic chest pain; d) myalgia; e) sore throat; f) headache AND illness not attributable to a non-infectious etiology. The components and single-case detection performance of the eight (8) ARI CDAs selected for the current studies are summarized in Table 1. CDA components included: a) provider-assigned ARI-related diagnostic codes (International Disease Classification, 9^th^ Revision, Clinical Modification, ICD-9) either used by the original Centers for Disease Control and Prevention (CDC) “BioSense” surveillance system [20] (the “CDC ICD-9 Codes” component in Table 1) or a code set previously adapted to the VA (“VA ICD-9 Codes”) [19]; b) prescription for cough suppressants (“Cough Remedies”); c) documented body temperature of ≥38°C; d) computerized analysis identifying at least two symptoms from the ARI case definition in the free text of the clinical note [19]. Time series of daily ARI counts were created for each CDA (those datasets are provided as Datafiles S1, S2, S3, S4, S5, S6, S7, and S8), and served as the backgrounds into which synthetic influenza epidemics were injected.

**Table 1:** Description of ARI CDAs.

Category	Subcategory	Case-Detection Algorithm Number							
		1	2	3	4	5	6	7	8
**CDA Components**	CDC ICD-9 Codes	•							
	VA ICD-9 Codes		•	•		•	•	•	•
	OR Cough Remedies			•			•		•
	OR Temperature ≥38°C								•
	OR Text of Clinical Note				•	•	•		
	AND Text of Clinical Note							•	•
**Performance**	Sensitivity (%)	63	79	85	88	97	99	69	75
		(57, 69)	(74, 84)	(80, 88)	(83, 91)	(94, 99)	(96, 100)	(64, 75)	(70, 80)
	Specificity (%)	92	97	96	93	90	89	99	99
		(91, 92)	(96, 97)	(95, 96)	(92, 93)	(90, 91)	(89, 90)	(99, 99)	(98, 99)
	PPV (%)	13	31	25	18	16	14	54	49
		(11, 15)	(28, 34)	(22, 27)	(16, 20)	(14, 18)	(13, 16)	(49, 59)	(44, 54)
	Area under the ROC	78	88	90	90	94	94	84	87
		(75, 80)	(85, 90)	(88, 92)	(88, 92)	(93, 95)	(93, 95)	(81, 87)	(84, 89)

Composition and performance of the eight (8) ARI CDAs used in this study. Individual CDAs are numbered in the first row. Black dot in columns 3–11 indicates that a component (column 2) is included in the corresponding CDA. Note that “performance” refers to ability to detect single cases with possible ARI. Performance numbers in parenthesis indicate 95% confidence limits.

#### Signal: synthetic influenza epidemic

**Figure 1:**
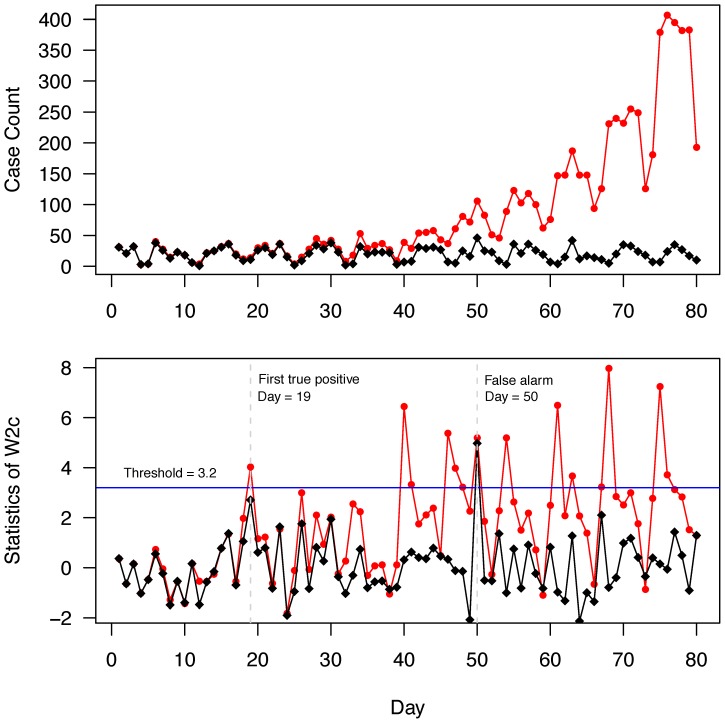
Simulated prospective surveillance cycle. Upper panel displays daily counts time series of authentic cases identified by CDA 2, either alone (*black diamonds*) or combined with simulated cases provided by the epidemic model for a community influenza outbreak that began at day zero (*red circles*). Lower panel shows the corresponding EARS W2c statistic for both time series (authentic cases alone (*black diamonds*) or combined with simulated epidemic cases (*red circles*)). True positive alarms occur when the value of the W2c statistic exceeds a threshold in the combined dataset while remaining sub threshold in the background dataset. For this 80-day surveillance cycle, at the arbitrarily set threshold of 3.2 (*blue horizontal line*), the time to the first true-positive alarm (detection delay) is 19 days. A false positive alarm occurs at day 50, when the statistic originating from the background-only dataset exceeds threshold.

To create a plausible ARI outbreak to be discovered by the surveillance system, we developed an epidemic model of influenza (Matlab R2008a, the Mathworks, Inc., Natick MA). The model included 30 contiguous ZIP codes centered on Baltimore, Maryland, and consisted of a coupled series of differential equations to describe the overall epidemic [21]. The susceptible population has the size and age structure described for Baltimore in the 2000 Census [22]. Age-specific death rates for each of the 20 demographic age-classes (age-class 1 = 0–4 years, age-class 2 = 5–9 years and so on) were derived from the United States Life Table Functions for the 1994 calendar year [23]. The birth rate for each age-class was obtained from Guyer *et al*. [24]. Model parameters were adjusted to mimic the estimated transmission and severity characteristics of the 1918 pandemic influenza in a non-immune population [25]. The proportion of cases that would be present for medical evaluation at the VA Maryland Health Care system was adjusted to reflect the age, gender, and population estimates of Baltimore veterans, over half of whom are older than 60 years old and more than 90% are male [26]. The same model-generated outbreak that was used as a common signal for all of our surveillance simulations is shown in Fig. 1 (upper panel, blue circles). Assuming that the synthetic epidemic cases would be discovered at the same rate as authentic cases, simulated cases from the epidemic model were first discounted by the sensitivity of the ARI CDA before being injected into CDA-specific background time series.

#### Surveillance simulations

We developed software aimed at replicating a surveillance system operating prospectively on the authentic historical background datasets described above (R v. 2.15.0, http://www.r-project.org). Starting on the day when the synthetic epidemic was injected into a CDA-specific authentic background time series, and then daily for a total of 80 days, a statistical outbreak detection method (see below) was applied in parallel to corresponding time series that included either: a) both background and epidemic cases (“Combined” dataset; Fig. 1, red circles, upper panel for the case counts, lower panel for the corresponding W2c statistic); or b) background-only cases (“Background” dataset; Fig. 1, black diamonds, upper panel for the case counts, lower panel for the corresponding W2c statistic). A true positive alarm was issued if the value of the computed statistic exceeded a set threshold in the Combined but not in the Background dataset (Fig. 1, lower panel, instances where red circles are above threshold whereas black diamonds remain below threshold). The 80-day cycle length was chosen because it ensured at least one true-positive alarm with each surveillance cycle. This prospective surveillance cycle was then repeated 51 times, each time shifting the outbreak injection to a different week of the one-year evaluation period (from 1/8/2002 to 31/7/2003, a year with average seasonal ARI activity).

#### Outbreak detection statistics

We used the “early aberration reporting system” (EARS) W2c [27], [28] statistical method to detect the injected epidemic. Daily case counts were separated a priori into two time series, one for weekdays and another for weekend and federal holidays. Using the appropriate time series for a given index day, the EARS W2c statistic is expressed as 
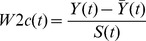
where 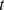 is the time series index, 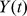 is the observed case count on that index day, 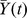 and 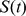 are respectively a 7-day moving sample mean and a standard deviation calculated with a 2-day lag from the index day. The value of 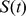 was replaced by 1 if 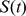
<1. The method signaled when the value of the W2c statistic exceeded a given threshold.

#### Performance measures

We computed three performance benchmarks at any given statistical alert threshold: 1) the Detection Delay, the time from the injection of the synthetic outbreak to the first true positive alarm, averaged for the 52 surveillance cycles of the evaluation year; 2) the false alarm rate (FAR), the average daily number of unique alarms issued in the background-only time series during the evaluation year; 3) the Caseload, defined as the total yearly number of cases included in the above false alarms. Corresponding Detection Delays, FARs and Caseloads were obtained for a range of statistical alert thresholds adjusted iteratively to focus on a FAR range felt to be of practical use for surveillance i.e. 0–10%.

## Results

**Figure 2:**
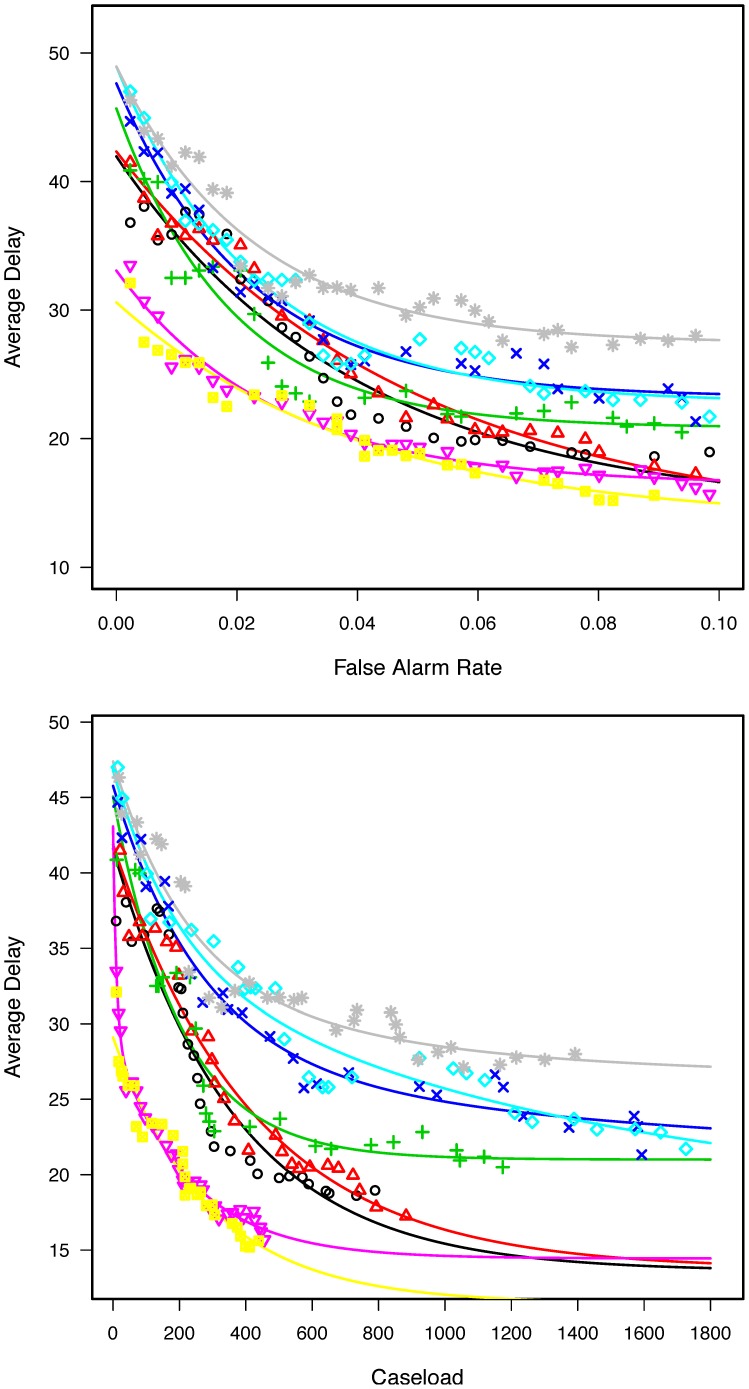
System performance using alternative case-detection methods. AMOC curves displaying epidemic Detection Delay (days) as a function of daily false alert rate (FAR) (*upper panel*) or yearly caseload (*lower panel*). Each curve represent an alternative CDA: CDA 1 (*grey stars*), CDA 2 (*black circles*), CDA 3 (*red triangles*), CDA 4 (*green crosses*), CDA 5 (*blue x's*), CDA 6 (*teal diamonds*), CDA 7 (*purple triangles*), CDA 8 (*yellow stars*).

The activity monitoring operating characteristic (AMOC) curves [29], [30] shown in Figure 2, upper panel, illustrate the relationship between average delay at detecting the synthetic influenza epidemic and the FAR for simulated surveillance systems that utilize one of eight (8) alternative case-detection approaches (Table 1, CDA 1–8).

### Effect of adjusting diagnostic code sets

The surveillance advantages of adjusting the ICD-9 codes used to identify ARI cases can be visualized by comparing the AMOC curves obtained using a “respiratory” code set used by a surveillance system of national scope [20] (Table 1, CDA 1; Fig. 2, upper panel, grey stars) with that of a surveillance code set adapted to the VA health system [19] (Table 1, CDA 2; Fig. 2, upper panel, black circles). Note that at any given FAR, CDA 2 resulted in shorter detection delay than CDA 1 (Fig. 2, upper panel, compare black circles with grey stars).

### Effect of combining diagnostic codes with text analyses of the clinical note

Case detection that relied solely on text analyses of clinical notes resulted in outbreak detection performance roughly on par with that of the VA-adapted set of ICD-9 codes (Fig. 2, upper panel, compare green “plus” signs (CDA 4) to black circles (CDA 2)). When combined to ICD-9 codes using an “OR” logical operand, text analyses boosted both case-detection sensitivity to 97% and the area under the ROC curve (Table 1, compare CDA 5 to CDA 2). Despite these performance gains, the [VA ICD-9 Codes OR text analysis of clinical note] case-detection approach worsened outbreak detection performance (Fig. 2, upper panel, compare blue “x” signs (CDA 5) to black circles (CDA 2)). In contrast, combining text analyses of clinical notes with ICD-9 codes using an “AND” logical operand, which improved specificity and PPV at the expense of sensitivity and lowered the area under the ROC curve (Table 1, compare CDA 7 to CDA 2), improved outbreak detection performance (Fig. 2, upper panel, compare purple triangles (CDA 7) to black circles (CDA 2)).

### Effect of adding selected structured EMR data other than diagnostic codes

We had previously found that selected structured EMR data, such as the documentation of fever or a prescription for cough suppressants, could further improve the test characteristics of ARI CDAs (Table 1, compare CDA 3 to CDA 2, CDA 6 to CDA 5, and CDA 8 to CDA 7) [19]. These improvements in case detection performance did not translate into improved outbreak detection (Fig. 2, upper panel, compare the following AMOC curve pairs: a) red triangles (CDA 3) to black circles (CDA 2); b) teal diamonds (CDA 6) to blue “x” signs (CDA 5); and c) yellow boxes (CDA 8) to purple triangles (CDA 7)).

### Effect of case-detection strategies on surveillance caseload

Figure 2, lower panel, illustrates AMOC curves of surveillance systems based on the ARI CDA shown in the Table 1, this time replacing the FAR by the corresponding number of yearly cases contained in these false-alarms. We have named this variable “caseload” because it reflects the amount of work (phone calls, records reviews) a public health practitioner would have to perform to investigate the system's false alarms in a given year. Replacing the FAR by its corresponding caseload upheld the utility of adjusting the diagnostic code sets (Fig. 2, lower panel, compare grey stars (CDA 2) to black circles (CDA 1)) and did not support the addition of structured EMR information about cough suppressants or fever (Fig. 2, lower panel, compare red triangles (CDA 3) to black circles (CDA 2); teal diamonds (CDA 6) to blue “x” signs (CDA 5); and yellow boxes (CDA 8) to purple triangles (CDA 7)). Caseload information further emphasized the advantages of coupling ICD-9 diagnostic codes with text analyses using an “AND” logical operand to identify ARI cases. To wit, under our experimental conditions, a public health department willing to investigate false alerts that involved 200 cases/year would discover the influenza outbreak in 20 days with CDA 8 (Fig. 2, lower panel, yellow boxes) compared to 33 days with CDA 2 (Fig. 2, lower panel, black circles).

## Discussion

We reconstituted a surveillance system in software to evaluate the impact of alternative EMR-enabled case-detection approaches on outbreak detection. Our data suggest that text analyses seeking ARI symptoms documented in ambulatory care visit notes can be combined with provider-assigned diagnostic codes to discover a modeled influenza epidemic sooner and to reduce the number of cases contained in false-alerts. These data support our working hypothesis that information harnessed from a comprehensive EMR can improve timeliness and efficiency of public health surveillance.

If simulated data have often been used to compare statistical approaches to cluster detection [28], [31]–[35], we could not find prior reports of the use of whole-system simulations to determine how case-detection methods affect outbreak-detection performance. The CDAs evaluated in this study were developed against a validated manual reference standard and only included EMR data elements found to contribute to ARI detection [19]. The CDAs were implemented against authentic EMR entries and thus produced backgrounds expected to mimic the noisy surveillance conditions found in the real world. In keeping with our goal to create a realistic evaluation platform, we also used an epidemic model to simulate the cases expected to present to our particular health system during an outbreak of severe influenza. Model-generated cases were sporadic at first but soon attained numbers large enough to be discovered by just about any surveillance method. Such an epidemic signal narrowed the time window over which alternative surveillance methods could demonstrate their superiority over one another.

Most automated surveillance systems utilize diagnostic codes to find diseased individuals. We [19] and others [36] have demonstrated that even minor adjustments of code sets to account for local practices can significantly strengthen case-detection performance. We now report that those same adjustments accelerated outbreak detection. Our data bolster the argument for providers to assign ICD-9 codes to summarize outpatient encounters, as it is done in VA medical centers, so that this coded information can quickly be made available for surveillance purposes. Our results further argue that diagnostic codes sets used for case-detection should routinely optimized during surveillance system development or expansion. In contrast, prescriptions for cough suppressants, the only structured EMR data found to contribute to ARI case detection aside from ICD-9 codes [19], did not benefit outbreak detection. While these data suggest how to streamline CDAs for this particular use-case, structured EMR data other than diagnostic codes may very well prove useful in discovering diseases that typically cause abnormal vital signs or prompt predictable diagnostic or therapeutic interventions.

Information potentially useful to public health surveillance has long been extracted from the free text narrative of chief complaints [37]–[41], laboratory or imaging reports [37], [38], [41]–[44], hospital discharge summaries [45], [46] or outpatient clinical notes [19], [47], [48]. To date however, little is known about how information extracted from free text EMR fields should be combined with structured data to accelerate outbreak detection. We had previously found that text analyses aimed at abstracting ARI symptoms typed in clinical notes [49] could improve the performance of ARI CDAs [19]. Our simulations suggest that when these text analyses were combined with structured data so as to improve case-detection accuracy, timelier outbreak discovery ensued. In contrast, when text analyses were directed toward maximizing the area under the ROC for single-case detection, outbreak detection performance deteriorated despite near-perfect case-detection sensitivity. These results seem counter-intuitive until we consider that outbreak detection depends upon a statistical rendering of the size of the epidemic signal relative to that of a baseline. With sensitivities in the 69–75% range, the high-specificity CDAs did not recognize the largest possible number of epidemic cases. Yet, with PPVs in the 50% range, they disproportionately reduced the number of false-positive background cases and improved the system's signal-to-noise ratio. Our results therefore suggest that CDA performance measures that attribute equal weight to sensitivity and specificity, such as the area under the ROC curve, may not anticipate how well a CDA will discover epidemics. Because of the complex interplay between epidemic signals, background noises and statistical processes, promising CDAs should be evaluated in a whole-system context before they are incorporated into an operational surveillance system.

## Limitations

Even though we expect the empiric approach to system improvement outlined this report to be broadly applicable, specific results are necessarily confined to our experimental conditions. Text analyses would indeed be expected to complement structured data differently for diseases that are not defined through symptoms only. Optimal EMR data integration may also depend on the characteristics of the epidemic, as well as on the nature and utilization of the EMR in other health care cultures. Our assumption that epidemic cases would be discovered at the same rate as authentic cases may also not hold in reality, as real epidemic cases may have a peculiar disease presentation or severity, and vigilant providers may change their coding or documentation behavior. Our results may also not represent the final word on ARI outbreak detection in our own health system, as we did not formally optimize individual system components, such as ICD-9 groupings, text analyses or statistical approaches, and have not validated our simulation findings in other medical centers.

## Summary

This work highlights an empiric approach to guide the integration of complementary EMR data for the purpose of epidemic surveillance. Through modular software development, a realistic evaluation platform can be harnessed to estimate the cost and benefit of alternative system configurations, or to match detection sensitivity with available resources to investigate alerts. The platform can also be extended to help improve detection of any disease or event cluster, and may thus allow automated surveillance systems to more vigorously participate in the promotion of safe and effective healthcare practices.

## Supporting Information

Datafile S1 **(CDA 1) Time series of background casecounts.** 8-year background time series are provided for each of the CDA outlined in the Table 1 (Datafile S1 (CDA 1), Datafile S2 (CDA 2), Datafile S3 (CDA 3), Datafile S4 (CDA 4), Datafile S5 (CDA 5), Datafile S6 (CDA 6), Datafile S7 (CDA 7), and Datafile S8 (CDA 8)). Each text file contains three columns: a) a line identification number (from 1 to 2921); b) the date ((m)m/dd/yyyy); and c) the corresponding case count returned by the CDA identified in the file title.
(TXT)

Datafile S2 **(CDA 2) Time series of background casecounts.**
(TXT)

Datafile S3 **(CDA 3) Time series of background casecounts.**
(TXT)

Datafile S4 **(CDA 4) Time series of background casecounts.**
(TXT)

Datafile S5 **(CDA 5) Time series of background casecounts.**
(TXT)

Datafile S6 **(CDA 6) Time series of background casecounts.**
(TXT)

Datafile S7 **(CDA 7) Time series of background casecounts.**
(TXT)

Datafile S8 **(CDA 8) Time series of background casecounts.**
(TXT)
